# Correlates of continuum of maternal health services among Nepalese women: Evidence from Nepal Multiple Indicator Cluster Survey

**DOI:** 10.1371/journal.pone.0215613

**Published:** 2019-04-19

**Authors:** Binaya Chalise, Mala Chalise, Bihungum Bista, Achyut Raj Pandey, Subash Thapa

**Affiliations:** 1 Nepal Health Research Council, Kathmandu, Nepal; 2 Nepal Institute of Health Sciences, Kathmandu, Nepal; 3 Research Unit of General Practice, Department of Public Health, University of Southern Denmark, Odense, Denmark; ESIC Medical College & PGIMSR, INDIA

## Abstract

Continuum of Care (CoC) is an essential strategy to prevent maternal and child deaths where health services are arranged in a pathway throughout pregnancy, childbirth and after delivery. However, CoC is still a challenge in Nepal. This study aimed to investigate the correlates of CoC from pregnancy to the postnatal period in Nepalese women aged 15 to 49 years. Secondary analysis was performed on the data from Nepal Multiple Indicator Cluster Survey. This led to a sample size of 2086 women who had a live birth within two years preceding the survey. We constructed three outcome models and conducted multivariable logistic regression, to assess socio-economic and demographic correlates of CoC from pregnancy to childbirth to postnatal period. Overall, 41% of the women received Antenatal Care (ANC), delivery from Skilled Birth Attendant (SBA) as well as the Postnatal Care (PNC) during their most recent birth. Women from rural areas (aOR 0.25, 95%CI: 0.18, 0.36) had reduced odds of receiving CoC while women belonging to advantaged ethnic group (aOR 1.61, 95%CI: 1.18 2.19), from middle wealth status (aOR 2.56, 95%CI: 1.68, 3.91) and upper (aOR 4.50, 95%CI: 3.07, 6.59) wealth status, and women having access to media (aOR 1.76, 95%CI: 1.31, 2.37) had higher odds of receiving CoC from pregnancy to postnatal period. Having more than two births reduced the odds of CoC by 30% (aOR 0.70, 95%CI: 0.50, 0.98). These factors were also significantly associated with ANC services and the continuum from ANC to delivery SBA. The findings suggest that the majority of Nepalese women lack a continuity of care during their pregnancy and childbirth, and several socioeconomic factors affect the spectrum of CoC. Efforts to improve maternal health services utilization in a continuum require strategies that remove demand and supply barriers of health care utilization.

## Introduction

Globally, Maternal Mortality Ratio (MMR) declined from 385 to 216 deaths per 100000 live births between 1990 and 2015 with the highest rate of decline occurring in developing countries [1]. Similarly, the under five mortality rate declined from 91 to 43 deaths per 1000 live births between 1990 and 2015 [2]. Maternal and under five mortality dropped substantially in South Asia with overall reduction of MMR by 68% and the under five mortality rate by almost 50% during this period [1, 2]. Consistent with the Global trend, maternal mortality in Nepal halved from 539 per 100000 live births in 2001 to 239 per 100000 live births in 2016, and neonatal mortality dropped from 50 to 21 deaths per 1000 live birth during the period [3]. Although the declines are a remarkable progress, neonatal and maternal mortality rates in Nepal are still the highest in the region. The trends imply that Nepal still lags behind in achieving health targets as stated in Sustainable Development Goals (SDGs) [4].

Most of the maternal and neonatal deaths are largely preventable in developing countries if maternal health services are provided in a Continuum of Care (CoC) approach [5]. The focus of CoC approach is to improve Maternal, Newborn and Child Health (MNCH) outcomes by integrating maternal and child health services on the time and place dimensions [6–9]. The time dimension of CoC specifices that maternal and child health services should be integrated and offered continually throughout the lifecycle including adoloscent, pregnancy, childbirth, postnatal period and childhood for improving survival and health of mother and newborn. The place dimension of CoC highlights and links services provided at home, communities and health facilities [6, 7, 9, 10].

For the purpose of this study, we have narrowed down the scope of CoC to the time dimension of continuity of three maternal health care services—Antenatal Care (ANC), Skilled Birth Attendant (SBA) and Postnatal Care (PNC)—during the period from pregnancy to childbirth to the time after delivery. The time dimension of CoC reflects need for optimum healthcare when the risk for complications to mother and baby is highest. For instance, more than half of all maternal and neonatal deaths occur during birth and in first few days of life, however, health care service utilization is lowest during this period [11]. Thus, information about the correlates of CoC in terms of time dimension of continuity of maternal health services is important.

The major advantage of a CoC approach is that each stage is profoundly intertwined, with the success of each stage building on the previous stage. For example, ANC is an important screening service that can assist in the prevention of problems during pregnancy and encourages women to opt for skilled provider assisted delivery [12]. ANC also offers an opportunity for other family members to become familiar with the health of women and motivate them to seek maternal health services [12–15]. Similarly, SBA during delivery and the subsequent PNC within 48 hours are overwhelmingly effective in reducing the risk of death and disability for the mother and newborn [16, 17]. An effective CoC links crucial MNCH services across the pregnancy, delivery, and postpartum stages while lack of appropriate health care services at any stage of CoC leads to poor MNCH outcomes [9]. Thus, the principle of CoC is, unequivocally, a key to achieving the targets of maternal and neonatal mortality as laid down in SDGs.

Statistics suggest that ANC (at least once by skilled providers) has significantly increased from 58% in 2011 to 84% in 2016, but only 69% of these women complete four ANC visits as recommended by the WHO. Similarly, only 58% of all deliveries are assisted by health workers and only 57% of women receive PNC within 48 hours after child births [3, 18]. This data is indicative that grave disparities exist in coverage of each of these maternal health services but this data is limited in quantifying the number of women seeking maternal health services along the pathway from ANC to PNC.

Understanding of the magnitude and factors that contribute to gaps across service use during antenatal, delivery and postnatal care is imperative for successful implementation of continuity of maternal health service and eventually for improvements in MNCH outcomes. A wealth of studies [19–22] investigated determinants of maternal health service utilization in Nepal. However, as the scope of these studies were an individual component of maternal health services- either ANC, SBA or PNC- they do not aid to our understanding of where and why do the gaps in seeking care along the pathway of CoC exists. Therefore, we considered ANC, SBA and PNC as an integrated maternal health care from the perspective of continuity over the time at different stages of pregnancy and childbirth to explore correlates of continuity of maternal health services. In doing so, we used data from the Nepal Multiple Indicator Survey (MICS), 2014 [23] to assess the socioeconomic and demographic correlates of women’s continuum of care from pregnancy to the postnatal period.

## Method

### Data source

We extracted data of individual women record from the Nepal MICS data file available from the Central Bureau of Statistics (CBS), Nepal. CBS, Nepal and UNICEF approved and oversaw the overall research process of the MICS. MICS micro-data is an openly accessible dataset that does not uncover the personal identity of the research participants. So, this study does not require ethical approval from respective institutions. The MICS is a nationally representative cross-sectional survey conducted by the Government of Nepal in 2014 to monitor health status of women and children in particular. The study was conducted before the massive earthquake in Nepal. Hence the findings might not be reflective of the most recent service use and its correlates, given the damage suffered by both the demand and supply side. A detail description of the MICS and its methodology is reported elsewhere [23].

### Sampling

The MICS identified rural and urban sampling strata from 15 sub-region reflecting the three Ecological and five Administrative regions of Nepal. The survey adopted a two-staged sampling design. The first stage involved systematic selection of 520 clusters with probability proportion to size from urban and rural strata. The second stage consisted of selecting 25 households per cluster through a systematic sampling from a household listing. 14162 women aged 15 to 49 years were successfully interviewed from the 12405 households out of which, our analysis was restricted to 2086 women who had live birth within two years preceding the survey. Our focus was on the health care services that they received for their most recent live birth.

### Study variables

ANC, SBA and PNC were the major outcome variables. ANC was referred to as health checks that a woman had, at least once, by a skilled provider (doctor, nurse or midwife) during her last pregnancy. Similarly, SBA was defined as delivery attended by a skilled provider during the most recent live birth of a woman. PNC was referred to as a health check-up by a skilled provider that a woman and her neonate had following delivery or a post-natal visit within two days after delivery. The independent variables were socio-demographic factors (woman’s age, ethnicity, religion, education attainment and woman’s access to media), household characteristics (education attainment of household head, family size and wealth index), contextual factors (place of residence and ecological region) and maternal factors (number of children ever born and history of child death). Ethnicity was based on the caste system in Nepal, which was broadly categorized as advantaged and disadvantaged ethnic groups based on the socio-cultural hierarchy in Nepalese society. Religion was broadly classified as Hindus and non-Hindus. Education attainment was defined as the primary or higher levels of schooling completed by the participants. Women were said to have access media if they read newspaper or listen to the radio or watch television at least once a week.

### Data analysis

We constructed three different outcome Models considering the continuum of care from pregnancy to childbirth and after delivery. The first Model focused on ANC as an outcome at which women receiving at least one ANC from skill providers were coded as “1” and “0” for those women who did not received ANC from skilled providers. In Model II, the outcome was receiving ANC as well as SBA from skilled providers. We thus coded “1” for women who received ANC and SBA from skilled providers and “0” otherwise. Women who received ANC, SBA and PNC from skilled providers were considered to have continuum of care in the third Model. The two categories of outcome for Model three were “1” for women receiving all three types of care from skill providers and “0” for otherwise.

Chi square test was used for bivariate analysis to assess association between the independent variables and the outcome Models. We used multivariable logistic regression in all three Models to identify correlates of the continuity of care from ANC to SBA and then to PNC. Covariates for the logistic regression were selected if the *p-value* was less than 0.25 in bivariate analysis. Multicollinearity among covariates was examined using Variance Inflation Factors (VIFs). Covariates with VIFs of more than 2.0 were excluded from the logistic regression analysis. We used STATA (version 15) and conducted complex survey analysis considering sample weight, Enumeration Areas (EA) as a primary sampling unit and place of residence (urban and rural) as strata for descriptive and regression analysis. We simultaneously adjusted for multiple variables in the models. The associations observed in this study are not due to confounding of any of the other variables in the models.

## Findings

### Background characteristics of women

Table 1 summarizes background characteristics of women included in this study. Most women (78%) had their recent births at age 29 years and below. Majorities (68%) of women were from disadvantaged ethnic groups and 84% were Hindus. Sixty-three percent of women had completed at least the primary level of education and reported having access to media. The majority of women were from lower economic background (43%) with family size of more than 4 members (72%). Eighty-seven percent of women lived in rural areas and 53% in the Terai.

**Table 1:** Characteristics of women who had live birth within two years preceding the survey.

	N = 2086	
	Un-weighted Count	Percent*
**Age of Women**		
15–29 Years	1621	78.5
30–49 Years	465	21.5
**Ethnicity**		
Disadvantaged	1155	65.8
Advantaged	929	34.2
**Religion**		
Hindu	1745	84.3
Non-Hindu	341	15.7
**Formal Education of Women**		
No	822	36.8
Yes	1264	63.2
**Women’s Access to Media**		
No	856	36.7
Yes	1230	63.3
**Formal Education of Household Head**		
No	883	44.3
Yes	1196	55.7
**Family Size**		
< = 4	605	28.0
> 4	1481	72.0
**Wealth Index of Family**		
Low	1205	43.4
Middle	311	21.5
High	570	35.0
**Place of Residences**		
Urban	343	12.8
Rural	1743	87.2
**Ecological Region**		
Mountain	616	7.2
Hill	741	40.4
Terai	729	52.5
**No of Births**		
< = 2	1353	67.2
>2	733	32.8
**History of Child Death**		
No	1786	88.4
Yes	300	11.6

*Sample weight was applied to calculate the percentage

### Overall use of maternal health services

Table 2 shows the combination of services that the women received for the most recent live birth. Twenty percent of women did not receive any maternal health services whereas 41% of women received all three types of services during their recent live birth. Only 7% of women received ANC and SBA but did not continue to receive the PNC. Around 17% of women receive at least one ANC from skilled providers but did not receive other two services. Similarly, few women received SBA (1.3%) or PNC (1.4%) or both (5.8%) without first having ANC.

**Table 2:** Different types of maternal health services received for the most recent births.

ANC Only	SBA Only	PNC Only	Frequency	Percent*
-	-	-	534	20.4
+	-	-	361	16.8
+	+	-	153	7.0
+	+	+	746	41.4
+	-	+	52	3.1
-	+	-	30	1.3
-	-	+	101	4.1
-	+	+	109	5.8
**Total Women**			**2086**	**100.0**

*Sample Weight was applied to calculate percentage

+ = Received Services,— = Did not Receive Services

### Continuity of care

Table 3 depicts facilitating or inhibiting factors for women to receive the three types of maternal health services during the child birth. Model I analyze correlates of using antenatal care. The result shows that place of residence is significantly associated with the use of ANC. For example, women living in rural areas had 73% (aOR 0.27, 95%CI: 0.15, 0.48) reduced odds of using ANC services whereas women from the Terai had 56% (aOR 1.56, 95%CI: 1.01, 2.43) increased odds of using ANC services. A woman from advantaged ethnic group had 66% (aOR 1.66, 95%CI: 1.19, 2.31) more odds of using ANC services than the women from disadvantaged ethnic group. The odds of using ANC were almost two (aOR 2.34, 95%CI: 1.59, 3.44) times and four (aOR 3.83, 95%CI: 2.49, 5.88) times higher among women from middle and upper wealth status households, respectively.

**Table 3:** Multivariable analysis of women who have received at least one anc visits from skilled providers.

	Model I*		Model II**		Model III***	
	aOR	95%CI	aOR	95%CI	aOR	95%CI
**Place of Residence** *(Ref*: *Urban)*						
Rural	**0.27**	**0.15, 0.48**	**0.19**	**0.13, 0.30**	**0.25**	**0.18, 0.36**
**Ecological Region** *(Ref*: *Mountain)*						
Hill	1.22	0.85, 1.73	**1.62**	**1.09, 2.42**	1.55	1.04, 2.30
Terai	**1.56**	**1.01, 2.43**	**2.00**	**1.24, 3.24**	1.40	0.86, 2.28
**Religion** *(Ref*: *Non-Hindu)*						
Hindu	1.31	0.89, 1.93	1.31	0.90, 1.90	**1.42**	**0.96, 2.10**
**Ethnicity** *(Ref*: *Disadvantaged)*						
Advantaged	**1.66**	**1.19, 2.31**	**2.05**	**1.50, 2.81**	**1.61**	**1.18, 2.19**
**Formal Education of HH** *(Ref*: *No)*						
Yes	1.17	0.88, 1.55	**1.42**	**1.07, 1.88**	1.09	0.81, 1.45
**Family Size** *(Ref*:*< = 4)*						
> 4	0.89	0.66, 1.20	1.04	0.75, 1.44	1.11	0.80, 1.52
**Wealth Index of Family** *(Ref*: *Low)*						
Middle	**2.34**	**1.59, 3.44**	**2.36**	**1.60, 3.54**	**2.56**	**1.68, 3.91**
Rich	**3.83**	**2.49, 5.88**	**3.97**	**2.69, 5.85**	**4.50**	**3.07, 6.59**
**Women Age** *(Ref*: *30–49 Years)*						
15–29 Years	1.10	0.79, 1.53	1.08	0.75, 1.54	1.01	0.70, 1.46
**Formal Education of Women** *(Ref*: *No)*						
Yes	1.19	0.88, 1.60	**1.45**	**1.06, 2.00**	**1.46**	**1.05, 2.04**
**Access to Media** *(Ref*: *No)*						
Yes	**1.40**	**1.07, 1.85**	**1.61**	**1.24, 2.11**	**1.76**	**1.31, 2.37**
**No. of Births** *(Ref*: *< = 2 Child)*						
>2 Children	**0.69**	**0.48, 0.97**	**0.57**	**0.41, 0.79**	**0.70**	**0.50, 0.98**
**History of Child Death** *(Ref*: *No)*						
Yes	0.80	0.54, 1.17	0.83	0.57, 1.20	**0.63**	**0.42, 0.93**

*I = Women who had at least one ANC visits from skilled providers (either Doctor, Nurse or Auxiliary Nurse Midwife) during their last pregnancy within two years preceding the survey.

**II = Women who had at least one ANC visit and delivery from skilled providers during their last pregnancy within two years preceding the survey.

***III = Women who had at least one ANC visit, delivery and Postnatal Care (at least one health checks) of mother and newborn within 48 hours of delivery from skilled providers during their last pregnancy within two years preceding the survey.

aOR = Adjusted Odds Ratio, CI = Confidence Interval, HH = Household Head

While women’s access to media increases the odds of using ANC services by 40% (aOR 1.40, 95%CI: 1.07, 1.85), having more than two births reduces the odds by 31% (aOR 0.69, 95%CI: 0.48, 0.97). Model II analyze the correlates of using ANC as well as SBA services. The findings indicate that all the factors having significant association in the first model also remained significant in the second model. In addition, the odds of using ANC as well as SBA was 45% (aOR 1.45, 95%CI: 1.06, 2.00) higher for women with primary or higher education than women with no education. Model III analyze effects of correlates on the continuity of care from delivery to the post-delivery period among women who had received ANC, SBA and PNC. All the factors found to be significant in Model II also remained significant in the third model. In addition, reduced odds were observed for women having history of child death (aOR 0.63, 95%CI: 0.42, 0.93) in the third model.

## Discussion

The Government of Nepal is explicitly focused on improving maternal and newborn health outcomes and the CoC has been one of its core strategies. It calls for an integrated health care system for providing ANC, SBA and PNC at all levels. However data on CoC is limited, which means, we are not able to precisely gauge where the country stands in terms of CoC, at which stage along the pathways of CoC do major dropouts occur and what factors might contribute this loss [6, 8]. The aim of this study was, therefore, to analyze the status of CoC in Nepal and further explore factors that affect women’s continuity of care from delivery to post-partum period.

This study demonstrated that 41% of the women avail continuity of care. This finding and evidence from another study [24] imply that more than half of the pregnant women in the country do not uptake all three maternal health services during the pregnancy and childbirth. We also observed that most of the dropouts occur between ANC and the use of SBA during delivery, which is substantiated by similar studies [6, 9, 25–27]. This might be due to home based deliveries, where nearly half of the deliveries in Nepal are conducted at home without SBA [3, 28]. The major factors contributing to home deliveries in Nepal are greater distance to health facilities and the lack of ANC visits during pregnancy. Studies [29, 30] suggest that women living farther than an hour away from a health facility are eight times, and women having no ANC visits are five times more likely to deliver at home. Like other studies [6, 8], most of the women who had SBA at delivery continued to receive postnatal services within 48 hours after delivery. This finding suggests that increasing the use of SBA during delivery could result in an increased uptake of postnatal care, and therefore, help to improve maternal and newborn health.

Our analysis indicated that women who were educated, belonged to advantaged groups, had a good family income and had access to media had higher odds of using at least one ANC visit, delivery and PNC from skilled providers during their last pregnancy. In contrast, women residing in rural areas and those giving birth to more than two children had less odds of continuing these services during their pregnancy and child birth. However, the observed significance for number of births should be interpreted with caution. We observed borderline significance for the variables, which could be just by chance due to descriptive nature of the study.

The study found a substantial discrepancy in continuity of care by place of residence, which is similar to other studies conducted in Cambodia, Pakistan, China and Ghana [6, 8, 9, 31, 32] Women residing in rural areas were less likely to utilize ANC, SBA and PNC services than their counterparts in urban areas as the ratio of hospital beds to population. The ratio of skilled providers (doctors, nurse and midwife) in rural Nepal is considerably lower than that for urban areas [33]. Moreover, women residing in rural areas face difficulties in accessing health services due to difficult geographical terrines, poor road network and lack of public transport [21, 34]. Consequently, fewer women in rural areas end up receiving continuous care. These factors raise awareness of the need for community based health care programs that address the underlying accessibility barriers to maternal and child health services. An important approach could be to provide ANC and PNC services to disadvantaged and rural women through home visits and outreach program [35]. Another strategy could be to include mass media campaign as key components of community based programs to improve continuity of maternal health care [36]. A recent Cochrane review [37] also showed that community based interventions such as outreach program and education campaign improve ANC coverage and number of skilled provider assisted deliveries.

Pregnant women in Nepal receive ANC, SBA during delivery and PNC at public and private health facilities. The Government has been implementing maternity interventions to improve the quality and continuity of MNCH services. These include, offering cash incentives to avoid transportation barriers to women who deliver in health facilities, providing free delivery care, providing cash incentive on first PNC visit for women who uptake a minimum of four ANC visit during pregnancy. In addition to this, newborn care is provided free of cost [28]. Despite the government’s effort to remove financial barriers, high dropouts between ANC and SBA during delivery implies that the focus should also be on removing supply side obstacles to health service utilization such as lack of drug supply and the availability of skilled health workers. Efforts should be aimed at addressing the supply side barriers thereby strengthening the capacity of health workers and health facilities to cater the health services need of pregnant women during and after delivery [38, 39].

The study found that level of education is a significant predictor of continuous utilization of maternal health services from pregnancy to postpartum period. This is in line with other studies [6, 34, 40–42] that women with higher educational level are more likely to utilize maternal health services. Another consistent finding is that women belonging to high income group and access to media had increased odds of utilizing maternal health services [6, 32, 40, 42]. On the other hand, parity had a negative effect on continuity of care. Studies [6, 34, 43] have shown that women having more number of children are less likely to continue to use maternal health services from pregnancy to postpartum period. Compared to primipara women, the perceived risk of pregnancy on health tends to be lower among high parity women [43]. As a result, high parity women may lack motivation to opt for ANC, and thus to use a skilled health worker during delivery and postpartum period. Further research needs to examine more closely the link between parity and women’s motivation to seek health services.

The study is subjected to some limitations. As the study is based on secondary data, we could not explore other factors such as obstetric complications that could potentially affect the use of maternal care services. The study is also limited by its inability to track ANC coverage by number of ANC visits, which would have given a comprehensive insight into continuity of ANC service and offer nuanced recommendations to reduce existing ANC discontinuity rate. Although MICS identified 520 clusters, one of these clusters was inaccessible due to high altitude and heavy snowfall during the time of survey thus resulting into selection bias. This does not, however, affect the generalisability of the findings, given the representativeness of included clusters as well as reasonable response rates of 98.5% for household response and 94.8% for women within interviewed households [23]. Moreover, measurement of outcomes is self reported based on face-to-face interview with women. This may have led to social desirability bias on the response they provide about the use of maternal health services resulting into misclassification and measurement errors. The study might not establish a causal relationship due to cross-sectional nature of the study.

## Conclusion

The study provides information about the socio-economic and demographic factors influencing the continuity of maternal health services among Nepalese women. For instance, poorer and uneducated women, who belonged to disadvantaged group and those residing in the rural areas are more likely to discontinue maternal health services during pregnancy and after childbirth. Community based health care programs are needed to address the supply and demand side barriers faced by the high risk women in utilizing maternal health services.
